# Intubation at Birth Is Associated with Death after Pulmonary Hemorrhage in Very Low Birth Weight Infants

**DOI:** 10.3390/children11060621

**Published:** 2024-05-22

**Authors:** Yong-Ping Sun, Hou-Bing Qin, Yun Feng, Yun-Su Zou, Yun Liu, Rui Cheng, Yang Yang

**Affiliations:** 1 Department of Neonates, Children’s Hospital of Nanjing Medical University, Nanjing 210008, China; 2 Respiratory Department, Children’s Hospital of Nanjing Medical University, Nanjing 210008, China

**Keywords:** pulmonary hemorrhage, postnatal tracheal intubation, preterm infants, mechanical ventilation, risk factor

## Abstract

Objective: This retrospective cohort study was performed to clarify the association between intubation in the delivery room and the mortality after pulmonary hemorrhage in very low birth weight infants (VLBWIs) during hospitalization. Methods: The study participants were screened from the VLBWIs admitted to the neonatal intensive care unit (NICU) of the Children’s Hospital Affiliated to Nanjing Medical University from 31 July 2019 to 31 July 2022. The newborns who ultimately were included were those infants who survived until pulmonary hemorrhage was diagnosed. These subjects were divided into the intubation-at-birth group (*n* = 29) and the non-intubation-at-birth group (*n* = 35), retrospectively. Results: Univariate analysis found that the intubation group had a higher mortality and shorter hospital stay than the non-intubation group (*p* < 0.05) (for mortality: 25/29 (86.21%) in intubation group versus 14/35 (40.00%) in non-intubation group). By multivariate analysis, the result further showed that intubation in the delivery room was related to shorter survival time and higher risk of death (adjusted hazard ratio: 2.341, 95% confidence interval: 1.094–5.009). Conclusions: Intubation at birth suggested a higher mortality in the VLBWIs when pulmonary hemorrhage occurred in the NICU.

## 1. Introduction 

In recent years, with the postponement of reproductive age and the development of assisted reproductive technology, premature births are increasing gradually in China. However, because of immature lung development, infants with small gestational age and low birth weight are prone to apnea, pulmonary hemorrhage, respiratory distress syndrome (RDS), bronchopulmonary dysplasia (BPD), and other respiratory diseases [1,2,3]. These infants often need invasive mechanical ventilation after birth. Among them, because of lower birth weight and smaller gestational age, very low birth weight infants (VLBWIs) are more vulnerable to pulmonary hemorrhage, which can cause respiratory failure, circulatory failure, shock, and even death [4].

For VLBWIs with poor birth status (where mask positive-pressure ventilation is unhelpful, or continuous positive-pressure ventilation and chest compression is needed), tracheal intubation is always an important intervention during postnatal resuscitation. In theory, intubation in the delivery room generally suggests the newborn has a weak spontaneous breath or severe pulmonary diseases (such as severe RDS, and congenital diaphragmatic hernia). Consequently, intubation seems associated with many adverse outcomes during hospitalization [5,6]. Meanwhile, it has been described that pulmonary hemorrhage is caused by many triggers. Asphyxia, prematurity, intrauterine growth restriction, infection, hypoxia, and patent ductus arteriosus (PDA) have been considered as perinatal risk factors for pulmonary hemorrhage by different researchers [7,8,9]. Moreover, pulmonary hemorrhage has also been reported related to tracheal intubation in the delivery room [10].

However, whether postnatal intubation is further related to the mortality risk in newborns with pulmonary hemorrhage is still unknown. In fact, few researchers have studied the association between postnatal intubation at birth and the death risk after pulmonary hemorrhage in premature babies. So, this retrospective cohort study was performed to further explore the connection between intubation at birth and mortality after pulmonary hemorrhage during hospitalization.

## 2. Patients and Methods

### 2.1. Preterm Infants

(1) Inclusion criteria: The newborns were screened from the VLBWIs admitted to the neonatal intensive care unit (NICU) of Children’s Hospital Affiliated to Nanjing Medical University between 31 July 2019 and 31 July 2022. The infants ultimately studied were VLBWIs who survived until pulmonary hemorrhage was diagnosed. 

(2) Exclusion criteria: Infants with severe hereditary metabolic diseases and congenital malformations were excluded. Patients who died before establishing the diagnosis of pulmonary hemorrhage were excluded. Neonates without complete data information in the case report form (CRF) were excluded, too. 

(3) Diagnostic criteria: Pulmonary hemorrhage was diagnosed as urgent blood secretion from the endotracheal tube that was associated with clinical deterioration, including increasing ventilation support with a fraction of inspired oxygen (FiO_2_) elevation of more than 0.3 from the baseline [7] or a rapid decrease in hematocrit (more than 10%) [11], as well as multi-lobular infiltrates on chest X-ray. RDS was diagnosed following the latest guideline—the 2019 European Consensus Guideline on the Management of Respiratory Distress Syndrome (ECGMRDS) [12]. BPD was graded according to a dependence on oxygen inhalation at 36 weeks PMA (NICHD 2001) [13,14]. Intraventricular hemorrhage (IVH) was graded following the research reported by Papile LA 1978 and Volpe JJ 2008 [15,16]. PDA and pneumothorax were defined according to Chinese Practical Neonatology [17]. Retinopathy of prematurity (ROP) was classified based on the papers published by the International Classification Committee on retinopathy of prematurity [18]. Sepsis was described based on the Chinese Expert Consensus on the Diagnosis and Management of Neonatal Sepsis [19]. Necrotizing enterocolitis (NEC) was defined based on the Bell’s stage [20]. 

(4) Exposure and grouping: The exposure factor was whether the baby was intubated when performing postnatal resuscitation in the delivery room. The subjects were retrospectively divided into two situations: intubation at birth (the intubation group) and non-intubation at birth (the non-intubation group). 

(5) Outcomes: The outcome we explored was the difference in mortality in VLBWIs with pulmonary hemorrhage between the intubation group and the non-intubation group. 

### 2.2. Clinical Data and Methods

(1) Clinical data: CRF data of all participants were collected by two doctors and checked by a third person. Data included maternal diabetes, maternal hypertension, prenatal glucocorticoid, amniotic fluid turbidity, mode of delivery, gestational age, birth weight, gender, Apgar score, blood gas, use of pulmonary surfactant, invasive and non-invasive mechanical ventilation, RDS, sepsis, BPD, medically and surgically treated PDA, IVH (≥grade III), NEC (≥stage II), mortality, and survival time during hospitalization.

(2) Postnatal resuscitation: Neonatal resuscitation of the VLBWIs was conducted following the Chinese Neonatal Resuscitation Guideline [21]. After birth, newborns were given positive and warm pressure ventilation with a mask, and then respiratory support was supplied by a T-piece. Whether there is a need for endotracheal intubation is determined by the doctor in the delivery room [21]. If mask positive-pressure ventilation is unhelpful or continuous positive-pressure ventilation by T-piece is needed or chest compression is conducted, then intubation is considered essential in the delivery room. After resuscitation, the patient was transferred to the NICU ward for further evaluation by the doctor on duty. Then, invasive or non-invasive ventilation was then adopted.

(3) Surfactant administration: The surfactant used in our ward is CUROSURF^®^, a naturally derived pulmonary surfactant produced by the Chiesi company, Parma, Italy. For each time, the dosage is 100–200 mg/kg of body weight. After birth, babies with positive end-expiratory pressure (PEEP) > 6 cmH_2_O and FiO_2_ > 30% would be given surfactant [22]. And, if there is evidence of the progression of respiratory distress, such as a sustained requirement for high-concentration oxygen and higher ventilation parameters, and other possibilities are excluded, repeated pulmonary surfactant is given [22]. 

When pulmonary hemorrhage occurred, the airway was fast cleaned by closed endotracheal tube suction, and then an effective ventilation strategy was adopted immediately. Since January 2017, our NICU has given infants an additional dose of surfactant after pulmonary hemorrhage by tracheal tube if the parents agree. The timing of surfactant administration is generally 2–4 h after pulmonary hemorrhage without evidence of active bleeding. The administration dosage is 100 mg/kg of body weight.

(4) Caffeine administration: The administration of caffeine was according to the 2019 ECGMRDS guideline [22]. The 2019 ECGMRDS guideline recommends 20 mg/kg as the loading dose after birth, then followed by 10 mg/kg daily for maintenance [22].

(5) Therapy for respiratory diseases: The indications for endotracheal intubation in NICU were determined by two criteria [20]. (1) Absolute indications: Partial pressure of carbon dioxide (PaCO_2_) more than 60 mmHg with persistent acidosis; Repeated apnea; Partial pressure of oxygen (PaO_2_) less than 50~60 mmHg with inhaled oxygen concentration more than 60%. (2) Relative indications: Severe dyspnea; Intermittent apnea; Blood gas analysis deteriorated evidently with PaO_2_ decreased and PaCO_2_ increased.

During the hospitalization, the attending doctor decided on the application of invasive ventilation [synchronized intermittent positive pressure ventilation (SIPPV), synchronized intermittent mandatory ventilation (SIMV), high-frequency oscillatory ventilation (HFOV)], non-invasive ventilation [nasal intermittent positive pressure ventilation (NIPPV), nasal continuous positive airway pressure (nCPAP), high-flow nasal cannula (HFNC), non-invasive high-frequency oscillatory ventilation (nHFOV)] and extubation. 

(6) PDA management: Echocardiographic measurements were screened for the first 24 h after birth and detected every few days using Vivid E9 ultrasound scanner (GE Company, USA). The measurements generally contain the ejection fraction, internal diameter of the duct size, pulmonary artery systolic pressure, end-diastolic reversal of blood flow in aorta, and left-to-right shunting of blood. For symptomatic PDA, moderate fluid restriction, proper mechanical ventilation strategy, and better circulatory perfusion were first conducted. Then, for PDA with ineffective conservative treatment mentioned above, ibuprofen was used for closing. If two courses of ibuprofen do not take effect, then surgical ligation is considered. 

### 2.3. Statistical Methods 

Statistical analysis was performed using SPSS 17.0 software. In terms of qualitative data, the Pearson Chi-square test or Fisher’s exact test was performed. Quantitative data that obey normal distribution were shown as mean and standard deviation. Comparisons between the two groups were performed using *t* or *t’* test. For skew distribution data, the median and interquartile range are used. Mann–Whitney test was used for comparison. For multivariate analysis, Cox regression analysis was used (Enter method). We assessed the potential confounding variables in our statistical modeling by the *p* value of previous univariate analysis and clinical experience. In this study, gestational age, birth weight, age at pulmonary hemorrhage, thrombin time, and grade III–IV RDS were included for Cox regression analysis. Crude and adjusted hazard ratio (HR) with 95% confidence interval (CI) were then collected. *p* < 0.05 was considered statistically significant.

## 3. Results 

### 3.1. Comparison of Perinatal History between the Intubation Group and Non-Intubation Group 

**Figure 1:**
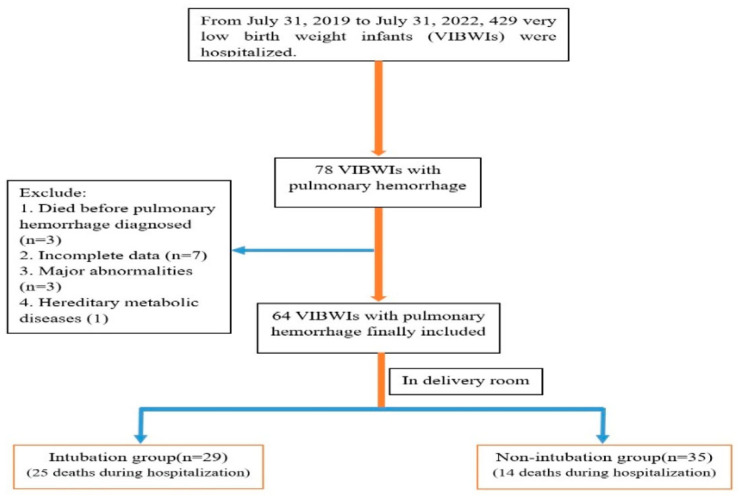
Flow chart of this study.

Between 31 July 2019 and 31 July 2022, 429 VLBWIs were hospitalized in our NICU, including 78 cases with pulmonary hemorrhage. A total of 64 VLBWIs were subsequently included in the study after screening by exclusion and inclusion criteria, containing 29 cases in the intubation group and 35 cases in the non-intubation group (Figure 1). The comparison showed that there were significant differences in birth weight, gestational age, Apgar score, and age at pulmonary hemorrhage between the two groups (*p* < 0.05) Table 1. 

### 3.2. Comparison of Ventilator Parameters, Blood Gas, and Coagulation Function before and after Pulmonary Hemorrhage between the Intubation Group and Non-Intubation Group 

(1) After 24 h of birth, the intubation group had a higher proportion of invasive mechanical ventilation, a higher fraction of inspired oxygen (FiO_2_) level, and a greater base excess (BE) value (*p* < 0.05). (2) The intubation group still showed a higher proportion of invasive mechanical ventilation prior to pulmonary hemorrhage (*p* < 0.05). (3) After pulmonary hemorrhage, higher peak inspiratory pressure (PIP) and longer thrombin time (TT) were found in the intubation group (*p* < 0.05) Table 2.

### 3.3. Comparison of Main Diagnoses and Therapies concerning Pulmonary Hemorrhage between the Intubation Group and Non-Intubation Group

In terms of main diagnoses, the intubation group had a higher proportion of grade III–IV RDS (*p* < 0.05). As for main therapies for respiratory diseases, the intubation group received more doses of surfactant before pulmonary hemorrhage and a longer duration of invasive ventilation during hospitalization (*p* < 0.05) Table 3.

### 3.4. Comparison of Main Outcomes between the Intubation Group and Non-Intubation Group 

The intubation group had higher mortality and shorter hospital stays compared with the non-intubation group (*p* < 0.05). But, there were no differences in other main outcomes, see Table 4. 

### 3.5. Cox Regression Analysis between Postnatal Intubation Resuscitation and Mortality after Pulmonary Hemorrhage

Variables including gestational age, birth weight, age at pulmonary hemorrhage, TT, and grade III–IV RDS were considered as covariates for further multivariable analysis. It was found that intubation in the delivery room was associated with a higher risk of death (adjusted HR = 2.341, 95%CI 1.094–5.009) Table 5.

## 4. Discussion

Contemporary incidence and clinical sequelae of neonates with pulmonary hemorrhage have not been well explored in the literature. Prior investigations of pulmonary hemorrhage have come from single-center studies with a small number of subjects. According to research from different NICUs, the incidence of VLBWIs varies from 0.5% to 11.0%, while the mortality is as high as 50–82% [23,24,25]. Our previous data found that the incidence of pulmonary hemorrhage in our NICU was 15.3% (42/275), and the mortality rate was 21.4% (9/42) in the population of VLBWIs [26]. 

Previous studies have mainly focused on describing the high risks related to pulmonary hemorrhage [23,24,25]. Among them, there is a tendency to believe that the occurrence of pulmonary hemorrhage seems to be associated with surfactant therapy for RDS in the past several years. In a meta-analysis of seven placebo-controlled studies using synthetic surfactant and four studies using animal-derived surfactant given either as prophylaxis or treatment of RDS, surfactant therapy was associated with pulmonary hemorrhage (risk ratio 1.47; 95% CI 1.05 to 2.07) indicating an increased risk for pulmonary hemorrhage with surfactant administration [27]. Even so, to our minds, the conclusion is still in doubt because pulmonary hemorrhage mostly occurs in preterm infants, especially in extremely preterm babies (refers to gestational age < 28 weeks). Those infants often have worse lung development and severe RDS (grade III or IV), which is also an important risk factor for pulmonary hemorrhage. So, to some extent, pulmonary hemorrhage is caused by severe RDS rather than surfactant replacement therapy itself. From our study, when compared with the non-intubation group, though the usage of surfactant is more common in the intubation group, it also showed that the incidence of grade III–IV RDS in the intubation group is significantly higher (Table 3). 

Intubation at birth has been elucidated to be closely associated with pulmonary hemorrhage. Wang TT et al. [10] recognized that the pulmonary hemorrhage group had a higher rate of intubation in the delivery room when compared with the non-pulmonary hemorrhage group (24/30 versus 77/130, *p* = 0.034). However, little is known about risk factors that are associated with survival in neonates with pulmonary hemorrhage. In the study from Ahmad KA et al. [4], significantly higher antenatal steroid exposure and cesarean section rates were identified in survivors of pulmonary hemorrhage. Compared with the decedents, the survivors showed a greater birth weight and a greater gestational age as well as a lower need for cardiopulmonary resuscitation in the delivery room. In our study, we also proved that the non-intubation group exhibited better birth weight and gestational age (Table 1). More importantly, infants without intubation showed older onset age, lower death risk, and longer survival time (Table 1 and Table 4). This, to some extent, further indicates that neonates with better developmental maturity also have a lower risk of mortality after occurring pulmonary hemorrhage.

In addition to the above, so far, limited research has studied the prediction model of death secondary to pulmonary hemorrhage. We believe that this study could provide a new insight for further large sample research concerning constructing the prediction model.

## 5. Limitations

We should note that the sample size of this study is not large enough. Moreover, the demographic data such as gestational age, birth weight, and other characteristics showed significant differences between groups, which suggests a certain bias in the interpretation of the conclusions. Some data including coagulation function after birth, and cardiac hemodynamics are not available because of the retrospective study design. Furthermore, the detailed process of cardiopulmonary resuscitation in the delivery room has not yet been fully obtained. Consequently, a better designed multicenter study is still necessary. 

## 6. Conclusions

Using univariate and multivariate analyses, we found that intubation at birth is associated with a higher risk of death in VLBWIs occurring after pulmonary hemorrhage during hospitalization. 

## Figures and Tables

**Table 1:** Comparison of perinatal history between the intubation group and non-intubation group.

Variables	Intubation Group (*n* = 29)	Non-Intubation Group (*n* = 35)	*χ*^2^/*t*/*Z*/*Fisher*	*p* Value
**Maternal hypertension** [*n*(%)] *	4 (13.79)	6 (17.14)	0.000	0.983
**Maternal diabetes** [*n*(%)] †	4 (13.79)	4 (11.43)	/	1.000
**Full course of prenatal glucocorticoid** [*n*(%)] #	7 (24.14)	8 (22.86)	0.014	0.904
**Amniotic fluid turbidity** [*n*(%)]	1 (3.45)	2 (5.71)	0.182	0.669
**PROM** > 18 h [*n*(%)]	6 (20.69)	8 (22.86)	0.044	0.835
**Cesarean section** [*n*(%)]	12 (41.38)	20 (57.14)	1.576	0.209
**Singleton** [*n*(%)]	17 (58.62)	19 (54.29)	0.121	0.728
**Birth weight** (Mean ± SD, grams)	988.97 ± 250.73	1144.00 ± 190.16	−2.740	0.008
**Gestational age** (Mean ± SD, weeks)	27.62 ± 1.57	28.93 ± 1.94	−2.930	0.005
**Male** [*n*(%)]	19 (65.52)	19 (54.29)	0.829	0.362
**Apgar score at 1 min** (Median + quartile)	7 (4, 8)	8 (7, 8)	−2.149	0.032
**Apgar score at 5 min** (Median + quartile)	8 (7, 9)	9 (8, 9)	−2.202	0.028
**Temperature at birth** (Mean ± SD, °C)	35.64 ± 0.70	35.66 ± 0.82	−0.108	0.915
**Intubation in delivery room** [*n*(%)]	29 (100.00)	0 (0.00)	/	/
**Age at pulmonary hemorrhage** (Median + quartile, days)	2.00 (2.00, 3.00)	5.50 (2.00, 12.25)	−3.011	0.003

* Maternal hypertension refers to hypertensive disorders during pregnancy. † Maternal diabetes includes diabetes in pregnancy and gestational diabetes. # Prenatal glucocorticoid use refers to the full course of treatment/(those who have not applied and have not completed the full course of treatment). Abbreviation: PROM: premature rupture of membranes.

**Table 2:** Comparison of ventilator parameters, blood gas, and coagulation function before and after pulmonary hemorrhage between the intubation group and non-intubation group.

Variables	Intubation Group (*n* = 29)	Non-Intubation Group (*n* = 35)	*χ*^2^/*Fisher*/*Z*	*p* Value
**24 h after birth**				
Invasive mechanical ventilation [*n*(%)]	29 (100.00)	28 (80.00)	4.621	0.032
Mechanical ventilation parameters				
PIP (Median + quartile, cmH_2_O)	19 (17, 22)	18 (16, 20)	−1.841	0.066
PEEP (Median + quartile, cmH_2_O)	6.00 (5.25, 6.00)	6.00 (5.00, 6.00)	−1.041	0.298
FiO_2_ (Median + quartile, %)	40.00 (40.00, 52.50)	35.00 (30.00, 40.00)	−2.532	0.011
Blood gas				
PH (Median + quartile)	7.28 (7.15, 7.35)	7.28 (7.23, 7.37)	−1.001	0.317
PaO_2_ (Median + quartile, mmH_g_)	72.00 (49.75, 96.75)	74.50 (43.25, 101.00)	−0.200	0.841
PaCO_2_ (Median + quartile, mmH_g_)	46.90 (34.00, 62.30)	51.65 (38.95, 57.88)	−0.117	0.907
BE (Median + quartile, mmol/L)	−7.90 (−10.20, −4.50)	−4.40 (−6.00, −2.70)	−2.825	0.005
PLT (Median + quartile, 10^9^/L)	204.00 (149.00, 248.00)	187.00 (168.00, 253.00)	−0.404	0.686
**24 h before pulmonary hemorrhage**				
Invasive mechanical ventilation [*n*(%)]	27 (93.10)	25 (71.43)	4.891	0.027
HFOV [*n*(%)]	1 (3.45)	0 (0.00)	/	0.453
Conventional mechanical ventilation parameters				
PIP (Median + quartile, cmH_2_O)	18.50 (17.00, 21.25)	18.00 (16.00, 20.00)	−1.225	0.221
PEEP (Median + quartile, cmH_2_O)	6.00 (5.50, 6.00)	6.00 (5.00, 6.00)	−1.503	0.133
FiO_2_ (Median + quartile, %)	35.00 (30.00, 42.50)	30.00 (26.50, 35.00)	−1.844	0.065
**After pulmonary hemorrhage**				
Invasive mechanical ventilation [*n*(%)]	29 (100.00)	35 (100.00)	/	/
HFOV [*n*(%)]	9 (31.03)	6 (17.14)	1.706	0.192
Conventional mechanical ventilation parameters				
PIP (Median + quartile, cmH_2_O)	23.50 (22.00, 25.75)	20.00 (20.00, 24.00)	−2.218	0.027
PEEP (Median + quartile, cmH_2_O)	7.00 (6.13, 7.75)	6.00 (6.00, 7.00)	−1.857	0.063
FiO_2_ (Median + quartile, %)	70.00 (50.00, 100.00)	80.00 (40.00, 100.00)	−0.485	0.628
Blood gas				
PH (Median + quartile)	7.03 (6.90, 7.10)	7.06 (6.85, 7.18)	−0.420	0.674
PaO_2_ (Median + quartile, mmH_g_)	48.50 (30.25, 84.75)	55.50 (40.00, 85.00)	−0.815	0.415
PaCO_2_ (Median + quartile, mmH_g_)	64.00 (52.43, 76.68)	75.80 (49.70, 88.10)	−1.045	0.296
BE (Median + quartile, mmol/L)	−13.70 (−18.20, −11.38)	−11.60 (−19.00, −4.83)	−0.810	0.418
Coagulation function				
PT (Median + quartile, seconds)	22.30 (17.70, 39.10)	19.70 (16.55, 25.53)	−1.414	0.157
APTT (Median + quartile, seconds)	90.45 (64.23, 141.73)	86.65 (59.63, 257.50)	−0.043	0.966
TT (Median + quartile, seconds)	20.05 (17.65, 24.48)	23.70 (20.30, 42.10)	−2.164	0.030
FIB (Median + quartile, g/L)	1.21 (0.92, 1.72)	0.91 (0.30, 1.45)	−1.957	0.050
PLT (Median + quartile, 10^9^/L)	116 (62, 171)	145 (99, 190)	−1.699	0.089

Abbreviation: PIP: peak inspiratory pressure; PEEP: positive end-expiratory pressure; FiO_2_: fraction of inspired oxygen; PaO_2_: partial pressure of oxygen in artery; PaCO_2_: partial pressure of carbon dioxide in artery; BE: base excess; PT: prothrombin time; APTT: activated partial thromboplastin time; TT: thrombin time; FIB: fibrinogen; PLT: platelet.

**Table 3:** Comparison of main diagnoses and therapies concerning pulmonary hemorrhage between the intubation group and non-intubation group.

Variables	Intubation Group (*n* = 29)	Non-Intubation Group (*n* = 35)	*χ*^2^/*Z*/*Fisher*	*p* Value
**Main diagnosis**				
Grade III–IV RDS [*n*(%)]	24 (82.76)	17 (48.57)	8.051	0.005
Medically and surgically treated PDA [*n*(%)]	20 (68.97)	19 (54.29)	1.436	0.231
Early onset sepsis [*n*(%)]	18 (62.07)	17 (48.57)	1.166	0.280
**Therapy for respiratory diseases**				
Use of surfactant before pulmonary hemorrhage [*n*(%)]	29 (100.00)	30 (85.71)	/	0.058
Dose of surfactant use before pulmonary hemorrhage (Median + quartile, times)	1.00 (1.00, 2.00)	1.00 (1.00, 1.00)	−2.487	0.013
Use of surfactant after pulmonary hemorrhage [*n*(%)]	4 (13.79)	7 (20.00)	0.104	0.747
Dose of surfactant use after pulmonary hemorrhage (Median + quartile, times)	0.00 (0.00, 0.00)	0.00 (0.00, 0.00)	−0.650	0.516
Duration of invasive ventilation (Median + quartile, hours)	76.00 (43.00, 172.50)	245.00 (74.50, 417.25)	−2.317	0.021
Duration of non-invasive ventilation (Median + quartile, hours)	435.00 (20.00, 829.50)	246.00 (122.75, 428.50)	−0.338	0.735
Course of invasive ventilation (≥3 courses) [*n*(%)]	1 (3.45)	6 (17.14)	/	0.116
Use of caffeine [*n*(%)]	29 (100.00)	35 (100.00)	/	/
Use of iNO [*n*(%)]	3 (10.34)	2 (5.71)	/	0.651

Abbreviation: RDS: respiratory distress syndrome; PDA: patent ductus arteriosus; iNO: inhaled nitric oxide.

**Table 4:** Comparison of main outcomes between the intubation group and non-intubation group.

Variables	Intubation Group (*n* = 29)	Non-Intubation Group (*n* = 35)	*χ*^2^/*Z*/*Fisher*	*p* Value
**Death** [*n*(%)]	25 (86.21)	14 (40.00)	14.225	<0.001
**Postnatal time of death** (Median + quartile, days)	3.00 (2.00, 5.50)	4.00 (1.00, 26.25)	−0.635	0.534
**BPD** [*n*(%)] *	5 (100.00)	20 (80.00)	/	0.556
**Severe BPD** [*n*(%)]	2 (40.00)	8 (40.00)	/	1.000
**Pneumothorax** [*n*(%)]	1 (3.45)	1 (2.86)	/	1.000
**NEC (≥grade II)** [*n*(%)]	3 (10.34)	10 (28.57)	3.255	0.071
**ROP** [*n*(%)] #	2 (6.90)	5 (14.29)	/	0.442
**IVH (≥grade III)** [*n*(%)]	12 (41.38)	7 (20.00)	3.473	0.062
**Length of hospital stay** (Median + quartile, days)	4 (2, 9)	45 (8, 65)	−3.365	0.001

* The proportion of BPD refers to BPD infants/infants who survived till 28 days after birth. # The proportion of ROP refers to ROP infants/all infants in intubation group or non-intubation group. Abbreviation: BPD: bronchopulmonary dysplasia; NEC: necrotizing enterocolitis; ROP: retinopathy of prematurity; IVH: intraventricular hemorrhage.

**Table 5:** Cox regression analysis between postnatal intubation resuscitation and mortality after pulmonary hemorrhage.

Variable	Crude HR	95%CI	*p* Value	Adjusted HR *	95%CI	*p* Value
**Intubation**	3.575	1.821–7.018	<0.001	2.341	1.094–5.009	0.028

Abbreviation: HR: hazard ratio. * adjusted by gestational age, birth weight, age at pulmonary hemorrhage, thrombin time, and grade III–IV RDS.

## Data Availability

The dataset used during this study is available from the corresponding author on reasonable request. The data are not publicly available due to privacy and ethical.
